# Esculapian Society

**Published:** 1863

**Authors:** George Ringland

**Affiliations:** Secretary pro. tem.


### ESCULAPIAN SOCIETY.

Abstract of the proceedings of the Esculapian Society of Wabash Valley.

The Society met at Marshall, Ill., May 27th, 1863. Dr. Charles Johnston was called to the chair.

Present, Drs. J. M. Steele, F. R. Payne, Johnston, Gorham, Holmes, Ringland, and Mitchell.

Drs. E. A. Steele, J. A. Patton, and R. F. Williams were elected members of the Society.

Dr. F. R. Payne read a short paper on the nature and treatment of corns. He urgently recommended the use of pure nitric acid as a reliable remedy for all varieties of corns and bunions. He does not scrape the corn, but applies the acid directly to the diseased surface every four or five days. It produces no pain, and gives almost instant relief.

Dr. Mitchell requested an expression of the members in relation to the nature and treatment of granular conjunctivitis. He believed it to be an idiopathic disease, caused by miasmatic influence acting on persons pre-disposed to scrofula. The granulations first appear, and, by their irritating influence, inflammation is produced and thickening of the membranes, resulting in opacity of the cornea. He learned from Dr. GROSS, while he was a resident of Louisville, that the most of his cases of this disease came from Wabash Valley.

Dr. STEELE remarked, that the physicians in the country could not treat cases of sore eyes with the same success as oculists, who make it a specialty, from the fact that patients are not constantly under their control and remedies could not be repeated as often as they should be. He believed that granulations were the result of inflammation. He had but little, in the treatment of the disease, but favored the use of anodyne and emollient applications as the main remedies.

Dr. PAYNE remarked, that the disease was one that had perplexed him for years. It was his opinion that the enlargement of the mucous follicles was the result of some grade of inflammation (either acute, sub-acute, or chronic,) of the conjunctiva. The irritation caused by these enlarged follicles produces inflammation of the cornea, thickening of its coats, and loss of sight. He believed, with Dr. MITCHELL, that this was more prevalent in miasmatic districts. He *had* treated the disease by cauterization and the knife for some years, but not with satisfactory results. His only treatment now was the use of a lotion composed of

Rose water,..........................................℥j.

Iodide of zinc,...............................g. ij. to vj.

applied freely to the ball of the eye every morning, and, during the balance of the day, he employs the following mixture:—

Rose water,..........................................℥j.
Morphine,..........................................
Sulph. zinc,.................................āā. gr. x.

applied frequently to the closed lids.

Dr. Ringland thought these granulations to be, generally, the result of severe and protracted inflammation. In some cases, especially where there are evidences of a scrofulous diathesis, very little inflammation precedes their formation.

In the great majority of cases, he thought very little benefit was to be derived from the use of strong caustics applied to the interior of the lids. He much preferred mild astringent and anodyne lotions, similar to those employed by Dr. PAYNE. If the irritation can be allayed, it will do much toward enabling nature to rid the parts of disease. Constitutional treatment should be employed where any unhealthy condition of system requires it; but it is often a purely local disease.

Dr. GORHAM said, he had but a limited experience in the treatment of this disease. His favorite remedy was

Rose water,........................................℥j.
Sulph. zinc,..............................grs. ij. to vj.
Morphine,..........................................v.

applied freely to the eye by means of a wide-mouthed bottle, (a morphine bottle answers the purpose very well). He instructs the patient to open and close the lids several times during the application, that the lotion may reach the whole of the diseased parts. In common inflammation of the eye, he had great faith in a solution of tartaric acid (v. to x. grs. to the oz.,) as a remedy. He deprecated the use of strong caustics to the eye.

Dr. J. M. STEELE had never treated a case of this disease. It does not prevail in his locality, so far as he knows. He has treated many cases of acute inflammation of the eye, which he generally arrested in a short time by free purgation with sub. mur. hyd., cupping the temples, and cold emollient applications. After the cathartic operates freely, he gives from twenty to thirty grains of quinine, divided into doses of three or four grains each. After the acute stage has passed, he uses a solution of acet. plumb. and opium.

Dr. Johnson had treated many cases of ophthalmia successfully, but his treatment of the granular disease was very unsatisfactory- His remedies were very simple. In the early stages, he required his patients to strictly observe a supine position; and he employed cold applications to the eye when the patient would bear them, otherwise, they should be warm. After the inflammation is somewhat reduced and there is a secretion of pus, he applies a solution of nit. arg. of a strength suited to the case. When an astringent is required, he prefers and infusion of cinchona and tannin.

Dr. HOLMES called for an expression of opinion in relation to the best method of treating puerperal peritonitis. Many cases had come under his care, and he was not satisfied with the result of his treatment.

Dr. J. M. STEELE was very confident his treatment of that disease was right, as it was so uniformly successful in curing his patients. He has treated many cases of it. After free purgation with sub. mur. hyd., with oil and turpentine at the onset of an attack, he mainly relied on the use of opium. In miasmatic regions, he would combine quinine with it for several days, in sufficient quantity to destroy the malarious influence; and, where a low typhoid grade of fever follows, he would continue it as a stimulant. If the tongue was dry and the secretions scanty, he adds to the opium three to six grains of sub. mur. hyd. daily. He moves the bowels every day with a mixture of two parts castor oil and one of turpentine, without stopping the opium. Large emollient poultices should be applied so as to cover the whole surface of the abdomen.

Dr. RINGLAND generally prescribed a mercurial cathartic at the commencement of an attack of this disease. His principal reliance was on opium. He gives it in sufficient quantity to produce a decided sedative impression. Where the patient is under miasmatic influence, he would combine quinine in moderately full doses for a day or two. He has frequently observed a decided improvement take place immediately after the operation of a second mercurial cathartic, administered after the continuous use of the opium for a few days. Warm emollient poultices are valuable adjuvants to the other treatment.

Dr. PAYNE had not treated a great number of cases of this disease. He agreed with Dr. STEELE. It was always his aim, after free purgation, to bring the patient under the influence of opium, and keep it up until the disease was arrested. If the pulse is not over 130, he feels confident this treatment will save the patient. He uses turpentine freely, both internally and externally. He prefers, for a poultice, the polygonum hydropiper, applied over the seat of pain.

Dr. J. M. STEELE inquired, whether any of the members had used the Bromine mixture in the treatment of erysipelas. No one had used it, but each gave his favorite treatment in this disease. Dr. PAYNE relied, principally, on the alcoholic tincture of iodine, locally, and the mur. tinct. iron, internally. Dr. J. M. STEELE'S favorite local remedy was the mur. tinct. iron. Dr. JOHNSON preferred a mixture of ung. hyd. one part, and lard three parts, applied to the diseased surface, covered with a heavy coat of very finely powdered starch,—much as he would treat a burn. Dr. MITCHELL considered the carb. lead or common white paint, the best local application.

The value of some new remedies were discussed. Dr. J. M. STEELE urged the members to give Bromine a fair trial in erysipelas.

Dr. PAYNE recommended tinct. arnica as a valuable new remedy in all bruises and sprains,—applied locally. He had employed tinct. aconite in many cases of neuralgia, and he regarded it as a good local remedy in that disease.

The Society adjourned; to meet at Grandview the last Wednesday of October.

GEORGE RINGLAND, *Secretary pro. tem.*

